# A brief intervention for weight management in primary care: study protocol for a randomized controlled trial

**DOI:** 10.1186/1745-6215-14-393

**Published:** 2013-11-19

**Authors:** Amanda Lewis, Kate Jolly, Peymane Adab, Amanda Daley, Amanda Farley, Susan Jebb, Deborah Lycett, Sarah Clarke, Anna Christian, Jing Jin, Ben Thompson, Paul Aveyard

**Affiliations:** 1 Department of Primary Care Health Sciences, University of Oxford, Radcliffe Observatory Quarter, Woodstock Road, Oxford OX2 6GG, UK; 2 Public Health, Epidemiology and Biostatistics, University of Birmingham, Edgbaston, Birmingham B15 2TT, UK; 3 Primary Care Clinical Sciences, University of Birmingham, Edgbaston, Birmingham B15 2TT, UK; 4 Medical Research Council Human Nutrition Research Unit, Elsie Widdowson Laboratory, Fulbourn Road, Cambridge CB1 9NL, UK; 5 Faculty of Health and Life Sciences, Coventry University, Priory Street, Coventry CV1 5FB, UK

**Keywords:** Obesity, Brief intervention, Weight management, Primary care, Commercial weight management services

## Abstract

**Background:**

Obesity affects 25% of the UK adult population but modest weight loss can reduce the incidence of obesity-related chronic disease. Some effective weight loss treatments exist but there is no nationally available National Health Service (NHS) treatment service, and general practitioners (GPs) rarely discuss weight management with patients or support behavior change. Evidence shows that commercial weight management services, that most primary care trusts have 'on prescription', are more effective than primary care treatment.

**Methods/design:**

We propose a controlled trial where patients will be randomized to receive either the offer of help by referral to a weight management service and follow-up to assess progress, or advice to lose weight on medical grounds. The primary outcome will be weight change at 12-months. Other questions are: what actions do people take to manage their weight in response to the two GP intervention types? How do obese patients feel about GPs opportunistically discussing weight management and how does this vary by intervention type? How do GPs feel about raising the issue opportunistically and giving the two types of brief intervention? What is the cost per kg/m^2^ lost for each intervention? Research assistants visiting GP practices in England (n = 60) would objectively measure weight and height prior to GP consultations and randomize willing patients (body mass index 30+, excess body fat, 18+ years) using sealed envelopes. Full recruitment (n = 1824) is feasible in 46 weeks, requiring six sessions of advice-giving per GP. Participants will be contacted at 3 months (postintervention) via telephone to identify actions they have taken to manage their weight. We will book appointments for participants to be seen at their GP practice for a 12-month follow-up.

**Discussion:**

Trial results could make the case for brief interventions for obese people consulting their GP and introduce widespread simple treatments akin to the NHS Stop Smoking Service. Likewise, the intervention could be introduced in the Quality and Outcomes Framework and influence practice worldwide.

**Trial registration:**

Current Controlled Trials ISRCTN26563137.

## Background and rationale

### Background

Obesity is an increasing problem in the UK, affecting around 25% of the adult population and the prevalence is projected to double in the next half century [1]. It is a major cause of morbidity and chronic disease, particularly increasing the risk of type II diabetes, cardiovascular disease and some cancers [2]. In addition, the direct economic cost in England is estimated at £4.2 billion per year [1]. There is good evidence that moderate weight loss (5 to 10% of initial body weight) among obese adults leads to beneficial clinical outcomes, particularly reducing the risk of diabetes [3]. However, there is a paucity of evidence on the effectiveness of the diverse range of available weight loss services [4], and whether these can be delivered at a population level through primary care.

We can use lessons learnt from tobacco control and apply these to obesity [5]. There are about nine million smokers in England. In 2009/10 English general practitioners (GPs)/family physicians made brief interventions to motivate cessation to about 3 million people, prescribed medication with brief support to 750,000, and another 750,000 attended the National Health Service (NHS) Stop Smoking Service [6,7]. The NHS Stop Smoking Service provides weekly behavioral support, either one-to-one or in groups, with medication to assist cessation. The UK GP pay-for-performance scheme, the Quality and Outcomes Framework (QOF), rewards GPs for recording smoking status of all patients and for intervening with patients with smoking-related diseases or diseases exacerbated by smoking (for example, asthma, diabetes). It is currently proposed to extend this so that GPs would be rewarded for intervening with all smokers, not just those with smoking-related disease. This activity is based on evidence from randomized controlled trials (RCTs) showing that GPs brief interventions motivate cessation [8], and that referral for more intensive behavioral support and medication enhances cessation rates over brief intervention alone [9,10]. Evidence from service evaluation of the NHS Stop Smoking Service shows that outcomes achieved are those seen in RCTs [11,12]. Taken together, brief interventions by GPs, prescribing of cessation medication, and referral to the NHS Stop Smoking Service make an important contribution to population cessation rates and reduced prevalence in the UK.

Keeping a register of all adults with obesity in a practice is now also a clinical management area of the QOF. However, GPs are required only to record weight and there are no indicators related to interventions, even in the case of obesity in diabetes. This reflects the lack of evidence from RCTs for opportunistic intervention and referral for weight management interventions that might be practicable in primary care. Unlike for smoking, GPs do not record whether or not they discuss weight management with their patients, there is no national surveillance system for recording brief interventions and there is no NHS weight management service, akin to the NHS Stop Smoking Service.

Two recent RCTs investigated practicable interventions for obesity management in primary care. In the Birmingham Lighten Up trial, 740 patients were randomized to one of six interventions or minimal control [13,14]. The interventions were 3 month programs with Weight Watchers (WW), Slimming World (SW), Rosemary Conley (RC), Size Down (an NHS group program), GPs and pharmacies. Dieticians trained GPs and pharmacists in weight management. Using intention-to-treat (ITT) analysis with (the conservative) baseline observation carried forward (BOCF), GP and pharmacy care achieved similar weight loss to the minimal control (1 kg at one year); that is, they were ineffective. However, those receiving WW, SW and RC lost 4 to 5 kg by 12 weeks, and at 1 year achieved 2 kg (SW and RC) to 3.5 kg (WW) weight loss. These results are similar to those from the trial by Jebb [15], where 772 participants randomized to 12 months WW or GP care achieved a weight loss of 4.0 kg and 1.6 kg, respectively, at 1 year. Over two-thirds of English primary care trusts (PCTs) currently contract with commercial weight management services (CWMS), costing PCTs about £50 per patient to provide a free (to the patient) 12-week treatment course ‘on prescription’. An earlier systematic review concluded that there was sufficient evidence of effectiveness for WW but not the other main CWMS in the US [16]. Thus, CWMS, and WW in particular, provide an evidence-based service analogous to the NHS Stop Smoking Service and to which patients can be referred in the NHS.

In considering the nature of a GPs brief intervention to induce weight loss in people who are obese, we examined several reviews [17,18] and considered data on current practice and the views of GPs on intervening [19-23]. A UK-based review recommended motivational interviewing (MI) as an evidence-based strategy to engage and support weight loss in patients in primary care. However, it noted that training in MI takes at least 2 days and that 15 minutes was the minimum time of counseling that has been found to be effective [17]. Physicians reported several barriers to intervening for weight management, but an important one was time constraints, with the average 10-minute GP consultation precluding use of MI. This means that current evidence-based MI interventions are unlikely to be widely implemented opportunistically and we therefore sought a complementary and briefer strategy. Other barriers reported include: lack of knowledge and lack of confidence to address obesity [20,21], and beliefs derived from anecdotal observations that weight loss interventions are unsuccessful [21]. The preferred intervention of GPs is brief directive counseling, often aimed at increasing awareness of health risks [21,22,24], an intervention not supported by evidence from randomized trials [17,18]. Another key barrier to intervention is fear that raising the topic of weight management opportunistically would offend patients [22]. However, a study showed that almost all patients who are obese recognize they have a weight problem, 95% want to lose weight, and 84% wanted help from their doctor to lose weight [25]. Patients who reported their doctor had helped them previously were more likely to have been referred to weight loss programs, had exercise recommendations made, or discussed the health risks (in descending order of helpfulness).

We also reviewed evidence of effective brief opportunistic interventions to reduce problem drinking and motivate smoking cessation [8,26]. It is clear from these reviews that genuinely brief (1 minute) interventions can motivate patients consulting for reasons unrelated to the behavior to either reduce their alcohol consumption or stop smoking. We recently reviewed the trials on physician advice for smoking cessation interventions, focusing on the key components of either advice to stop smoking on health grounds or offer of support on how to quit [27]. We found that interventions that offered support were more effective at initiating attempts to quit (as well as supporting abstinence) than was advice to quit on health grounds. We concluded that patients are more likely to take action if given an effective means to achieve their goal. This would be predicted by PRIME theory, where behaviors are the product of momentary desires which are in turn the product of stimulus-induced images of possible futures to which people feel attracted [28]. The Department of Health built on this evidence to produce a 30-second intervention for cessation: the 3As, ask (about smoking status), advise (to stop), and act (referral for cessation support). We propose an analogous intervention in weight management; that is, we propose a 30-second intervention that concentrates on referral to an evidence-based weight management service available currently in most of the NHS. In this protocol, we call this an assistance-orientated intervention and the traditional approach of raising the health risks we call an advice-orientated intervention.

We therefore sought evidence that genuinely brief interventions by GPs might motivate weight loss in people who are obese. The US Behavioral Risk Factor Surveillance System found that 42% of obese patients in 1996 reported that their physician had advised them to lose weight (ever) [19,23]. People who had received advice were more likely to have tried to do so (odds ratio (OR) 2.79 (2.53 to 3.08). By 2006 to 2008, 66% of obese patients had been told they were overweight by their physician [29]. Again, being told they were overweight was strongly associated with attempting to lose weight in the previous 12 months (OR 2.51 (1.74 to 2.88)). These data support the hypothesis that GP even advice-orientated brief intervention motivates weight loss, but bias or confounding could explain the association of recall of advice and attempts to lose weight.

To our knowledge there are no previous RCTs to assess the effectiveness of primary care led brief opportunistic interventions to support weight loss among people who are obese and therefore propose the current trial to assess an assistance-orientated intervention. This is based upon evidence that referral to CWMS operating within the NHS are effective, that suggesting referral takes only a few seconds, that data from analogous brief interventions on other behaviors suggest offering support is the most effective strategy, and there is some evidence to suggest that brief weight loss advice from physicians could be effective. In addition to referral to weight management services, we propose that GPs offer patients in the intervention group a follow-up appointment about a month after the initial consultation. First, this signals to the patient that the GP takes the issue of weight loss seriously and this may prompt a person to take action. Second, weight monitoring seems to be an important component of effective weight loss interventions [30]. Third, we found in the Lighten Up trial that some participants initially accepted a referral for weight management but then did not take that up until they were followed up, when a second referral was made. Fourth, English NHS guidance suggests using orlistat in patients who fail to lose weight despite adhering to an appropriate weight loss intervention. In practice, prescription of this pharmacotherapy is uncommon and we expect it to be so in this trial. Finally, we will give GPs minimal training to pass on a few tips on weight management that might be helpful. We therefore propose to test an intervention of several elements and it will not be possible to isolate the effectiveness of individual components. What we are proposing testing is brief opportunistic intervention and active engagement of GPs in supporting weight loss in patients who are obese.

We also considered the nature of a comparison intervention in the control arm. The most common response towards a person with weight problems in primary care is not to discuss this [22,24]. A reasonable comparator would therefore be no intervention. On the other hand, asking a person to participate in a trial related to weight management and offering no intervention makes it clear that a person is in the control arm. This could lead to differential follow-up, which would threaten validity. In the Lighten Up trial, people were more likely to agree to follow-up weighing when a person had lost weight than when they had not. The second most common response GPs make is to advise that weight loss would benefit health - an advice-orientated intervention [22,24]. We believe that offering advice to lose weight on health grounds to the control group does not reveal the random allocation to the participant and may assist follow-up. The Lighten Up trial showed that CWMS achieve higher weight loss than participants who try to lose weight unassisted and therefore there is a reasonable prospect of detecting a difference in effect between our advice-orientated control intervention and our assistance-orientated active intervention.

### Rationale for the current study

Tobacco control interventions have successfully reduced the prevalence of smoking in many societies. An important component is opportunistic interventions by the GP, which are known to be effective in smoking and problem drinking but not in obesity management. A recent systematic review identified no trials that examined whether screening to identify overweight or obesity in adults and brief intervention was effective [31]. This trial, the first to our knowledge, will test whether opportunistic weight management interventions are effective.

The intervention we propose makes only very modest demands on a GPs time and skills, which is a strategy we have chosen deliberately. If successful, and if implemented widely, it will increase the degree to which GPs engage with weight loss and weight maintenance and this may provide a base on which to build more complex interventions. Further, the trial results could make the case for brief opportunistic interventions for obese people consulting their doctor and introduce widespread simple treatments akin to the UK Stop Smoking Service. If brief interventions are effective, this would make a strong case to add brief interventions to the weight management component of the QOF. It may influence practice worldwide, as has been the case with brief interventions for smoking cessation.

### Objectives

#### Primary objective

1) To examine the effect of an assistance-orientated (brief referral and review) versus advice-orientated intervention (enhanced usual care) on mean weight change at 12 months.

### Secondary objectives

1) To examine the effect of an assistance-orientated versus advice-orientated intervention on mean weight change at 3 months *(*self-reported weight measure*).*

2) To examine the difference in the proportion of participants in each intervention group who achieved 5% and 10% weight loss at 12 months.

3) To examine how obese patients feel about discussing their weight with their GPs when they have visited for reasons other than their weight and how this may vary by intervention type.

4) To examine what actions people take to manage their weight, at 3 and 12 months, in response to the two types of GP intervention.

5) To examine how GPs feel about raising the issue of weight management opportunistically and giving the two types of brief intervention both before and after giving opportunistic interventions.

6) To assess the cost per kg, and per kg/m^2^, of the weight lost to the NHS for the two types of interventions.

## Methods and study design

### Summary of study design

#### Study design

A pragmatic RCT to compare 912 obese adults attending their GP for reasons other than weight management who receive an assistance-orientated intervention, with 912 of the same type of participants randomized to a control group who receive an advice-orientated intervention.

#### Study duration

**Figure 1:**
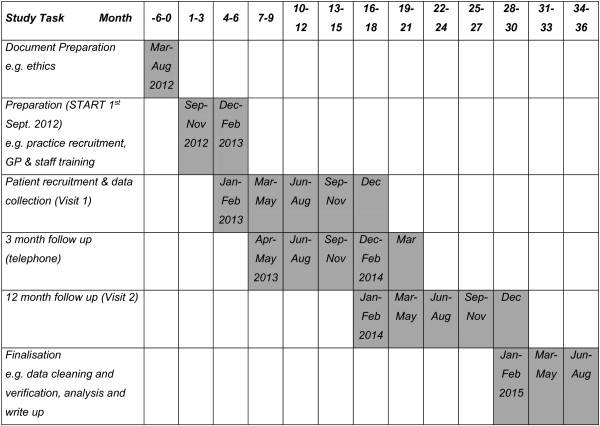
**Sequence and duration of all study periods.** GP, general practitioner.

The total study duration will be 36 months. The timeline (see Figure 1) illustrates the sequence and duration of all study periods.

#### Participant participation

**Figure 2:**
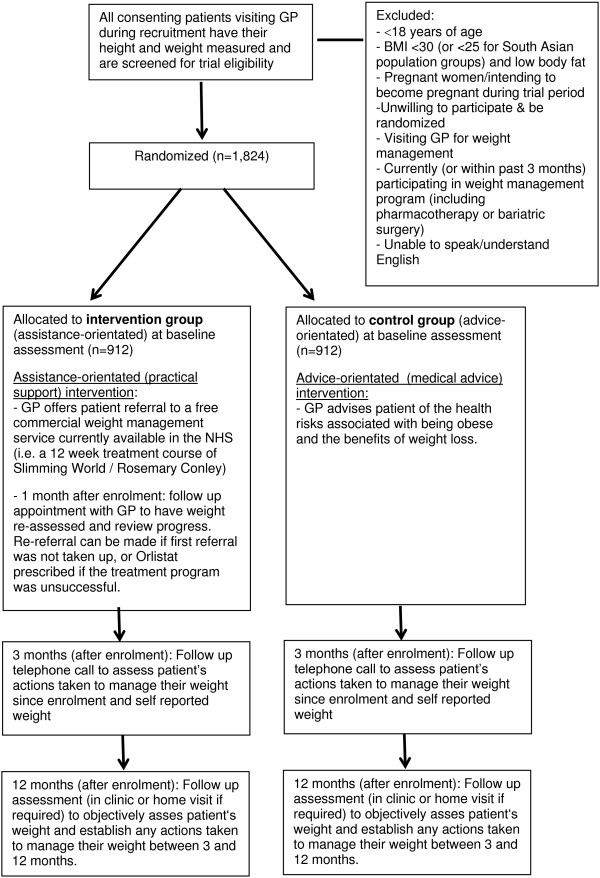
**Study flow chart.** BMI, body mass index; GP, general practitioner; NHS, National Health Service.

Each participant will be enrolled on the day of recruitment and followed up for 12 months (see Figure 2 for the study flow diagram).

#### Research sites

Sixty sites (that is, GP practices) within England. Additional sites may be added if necessary to meet recruitment targets.

### Primary and secondary endpoints/outcome measures

We will assess the following outcomes in response to the trial objectives outlined in section 2:

#### Primary outcome

1) Participants' weight change from baseline to 12 months*.*

#### Secondary outcomes

2) The mean change in participants' weight from baseline to 3 months.

3) The proportion of participants who lose 5% and 10% of their initial weight at 12 months.

4) Costs of the two types of intervention to calculate the NHS cost per kg, and per kg/m^2^, lost.

#### Non-efficacy outcomes

5) Participants' reaction to the GP discussing weight management, immediately after their GP appointment, when they visited for reasons other than their weight.

6) The actions people take to manage their weight, at 3 months, in response to the two types of GP intervention. This will include: attendance at the GP to discuss weight; whether participants still continue to manage their weight and the means they are using to do so; or if they have abandoned a weight management plan and why.

7) The actions people take to manage their weight between 3 and 12 months following a brief opportunistic intervention.

8) Participants’ thoughts about the opportunistic intervention that was delivered and whether other styles may have resulted in a different reaction than the one experienced.

9) GPs reactions to, including views on the helpfulness and appropriateness of, raising the issue of weight management opportunistically and giving the two types of brief intervention.

10) GPs thoughts about the brief intervention. This will include exploring: why GPs felt as they did; whether other brief interventions might be more or less acceptable than the one they gave; and whether these interventions might be suitable for all overweight people, or only those with relevant medical conditions.

11) GPs attitudes to making opportunistic interventions and treating obese people before and after participating in the trial.

#### Measurements of outcomes

This section outlines the different stages of the study, including how and when we will measure the above outcomes.

##### 

**Visit 1 – Baseline** There are three key purposes of this visit. First is the identification of potential participants, which is detailed below. At this point, we will ask to record personal information from all patients, primarily to assess eligibility for the trial, but to also update medical records as an additional benefit to the GP practice (Outcomes #1-3). Data to be collected by a research assistant during this initial visit (pre-general practitioner consultation) are as follows:

Form 1 - BWeL Trial Screening Form (These data are to identify potential participants; the research team will ask to keep a copy of this if a person is eligible for the study but declines participation. This is to compare the characteristics of people who participate to those who do not. In addition one copy will be passed to the GP to update records).

• Name (to identify the record) *Note: The research team will not keep this data for anyone who is eligible but not willing to participate in the trial, in line with the Data Protection Act, 1998.*

• Date of birth (to establish age and for identification)

• Main language spoken ([optional] for trial purposes and to update GP record)

• Ethnic group ([optional] for trial purposes and to update GP record)

• Height, weight and percentage body fat (to assess eligibility for the trial and to constitute baseline measures if so, and to update GP medical records)

• Gender

Form 2 - Written evidence of consent (for eligible and willing patients only).

Form 3 - BWeL Participant Contact Sheet (These data will only be collected for patients who are eligible and consent to be part of the BWeL trial).

• Address, including postcode

• Telephone number(s)

• Email address(s)

The second key purpose of this visit is to invite eligible patients to join the trial. If a patient is willing to participate, written consent to participate in the trial and randomization will take place immediately, as detailed below.

The third purpose is to measure participants’ reactions to the GP discussing weight management, immediately after their GP appointment, when they visited for reasons other than their weight (Outcome #5). The research assistant (RA) will ask all participants, upon exit of their appointments, for their randomization card and envelope (and withdrawal card if applicable). The randomization card will ask participants to rate the helpfulness and the appropriateness of the GPs very brief intervention on five-item Likert-type scales that the GP and/or RA will ask the participant to complete; this will be carried out confidentially in the RAs room. Where patients have not been randomized, the RA would explain that the GP does not feel it is suitable for them to be involved in the trial at this time and, therefore, they have not been enrolled; GPs will be asked to record why the patient was not randomized (see below).

##### 

**Follow-up telephone call at 3 months (after enrolment)** A researcher will contact all participants via telephone (or by alternative methods such as email, post or text message if telephone contact is not possible) 3 months after their initial appointment to assess what actions they have taken, if any, to manage their weight since the appointment (Outcome #6). We will also assess if participants are continuing to manage their weight and through which methods. If they are no longer managing their weight, we will ask why and record this on a simple checklist of possible reasons. We will ask participants to stand on their scales and read out their weight or report a recently obtained weight (Outcome #2). We will also ask how many contacts participants have had with their GP where the prime purpose was weight management. Pregnancy status will also be updated to ensure weight data are valid.

##### 

**Visit 2 - follow-up appointment at 12 months (after enrolment)** A researcher will telephone all participants to arrange for them to meet with an RA, for a 12-month follow-up appointment, at their own GP surgery. The main purpose of the visit is to objectively measure the participant’s weight (Outcomes #1 and 3). As a precautionary measure (that is, in case the participant does not attend their clinic follow-up appointment, or if participants decline to attend), during the booking telephone call the researcher will collect data on whether and what methods of weight management the participants have used/continued to use since the 3 month follow-up (Outcome #7). In addition, we will ask participants to stand on their scales and read out their weight or report a recently obtained weight. Pregnancy status will also be updated to ensure weight data are valid.

As the follow-up appointment holds no therapeutic value, we will pay those participants who attend a small reimbursement (£10.00) to cover the cost and inconvenience of attending.

##### 

**Before GPs have started their trial involvement** We will ask all GPs to complete a questionnaire containing a few short questions about their attitudes to making opportunistic interventions and treating obese people (Outcome #11).

##### 

**After GPs have finished their trial involvement** We will ask all GPs to complete a short questionnaire about their attitudes to making opportunistic interventions and treating obese people, using the same questions as used ‘before the GPs trial involvement’ (Outcome #11). In addition, we will ask about their views of the appropriateness and helpfulness of giving brief interventions (Outcome #9). We will subsequently briefly interview, via telephone, up to 30 participating GPs to examine their thoughts about the brief intervention (Outcome #10); GPs will be purposively selected based on the range of responses to the post-trial questionnaire to represent a range of reactions to delivering the intervention. The interview will explore why GPs felt as they did and whether other brief interventions might be more or less acceptable than the one they gave and whether these interventions might be suitable for all overweight people or only those with relevant medical conditions. We will contact selected GPs as soon as possible after receiving their questionnaire to minimize forgetting.

##### 

**After completion of participant recruitment** Similar to above, we will interview, via telephone, up to 30 participants based on the range of responses to the post-consultation questionnaire (see Visit 1) and ask them about why they responded as they did and whether another style of opportunistic intervention might engender a different reaction (Outcome #8). We will contact selected participants as soon as possible after the consultation to minimize forgetting.

##### 

**After trial completion** Costs of the two types of intervention will be assessed to calculate the NHS cost per kg, and per kg/m^2^, lost (Outcome #4).

#### Loss to follow-up

We will make a maximum of three attempts to contact participants before abandonment, using multiple means and trying different times. More than this could be seen as harassment. If participant’s data is missing we will use ITT analysis with BOCF for the primary outcome assessment (see below – Description of Statistical Methods).

### Study participants

#### Overall description of study participants

In total, 1,824 obese adults in England attending their GP for reasons other than weight management; 912 in each of the two treatment groups (see Sample size calculations below).

#### Inclusion criteria

Any patient, during recruitment, that:

• Is identified with a body mass index (BMI) ≥30 (or ≥25 for South Asian population groups) and excess body fat.

• Is ≥18 years of age.

• Consents to participate and comply with study procedures.

#### Exclusion criteria

The participant may not enter the study if any of the following apply:

• Pregnant or intending to become pregnant during the trial period (that is, the next 12 months).

• Currently or within the past 3 months participated in a weight management program (including pharmacotherapy or bariatric surgery).

• Unable to understand and speak English sufficiently to give informed consent and complete the research assessments.

• Visiting the GP for weight management.

• The GP deems it inappropriate to make an opportunistic intervention on weight management. This includes personal medical reasons known to the GP, such as an eating disorder, or reasons related to the consultation (for example, the patient has become distressed and it would seem insensitive to make such an intervention at that time). If the GP does not consider it appropriate, then the patient will not be enrolled and randomized into the trial (see Randomization of participants below).

### Study procedures

#### Informed consent

The Recruitment of participants section below specifies who will take informed consent and how and when it will be taken.

#### Study assessments

The Measurement of outcomes section above outlines the different stages of the study, including each assessment and time points. The sections below outline additional study procedures (that is the recruitment of GP practices and participants and the randomization, enrolment and withdrawal procedures).

#### Recruitment of GP practices

We will invite practices to participate without restriction as to the type of practice.

#### Recruitment of participants (including screening, eligibility and baseline assessment)

The trial will involve about 60 GP practices within England, where we propose recruiting our participants. On arrival at the practice, the receptionist will provide a participant information leaflet (PIL) to each patient attending for a GP consultation during that recruitment session. The PIL would inform patients that everyone visiting a GP during that session will be asked, by a RA before their appointment with the GP, if they (that is the RA) could briefly take a few measurements to update the practice medical records (as identified in Visit 1 - Baseline, Form 1, above). The PIL will also state “This is part of a study that is taking place at the practice; the researcher will tell you more. The researcher will also ask for your name, date of birth, ethnicity, and the language you speak. This will be done in a private consultation room and take less than 10 minutes. If you do not want to see the researcher, you do not have to. It will not affect the care we give you in practice today or in the future”. In practices where patients check-in for their appointment via an automated computer system upon arrival, rather than seeing a receptionist, the PILs will be positioned by the check-in system, and the on-screen message will be programmed to ask the patient to take one.

To check the patient’s health, the RA will measure height and weight and percentage body fat of willing patients in a vacant consultation room using medically approved and validated weighing scales (Tanita portable body composition analyser, model SC 240 MA); the RA will ask participants to remove excess clothing (for example, coats and large jumpers) and shoes to increase accuracy of body weight, body fat and height. On the advice of our GP consumer group, we will ask receptionists booking patients for this session to advise patients that this health check will be taking place and ask them to attend about 10 minutes prior to the appointment if possible. This is designed to help prevent disruption to the GP appointment schedule and provide reassurance to the patient that they have time to speak with the RA and will not miss or delay their appointment.

During the height, weight and body fat assessment, the RA will collect socio-demographic data (see Measurement of outcomes [Visit 1, Baseline] for details), so that the research team can examine whether the population enrolled were a biased selection of all those attending and, as a secondary benefit, to update the GP records. The RA will invite patients meeting the trial eligibility criteria (see Inclusion and Exclusion criteria above) to participate “in a study about people’s reactions and response to GPs discussion of their weight”; the RA will explain the trial to interested patients and answer any questions that they may have, as well as provide them with a trial patient information sheet (PIS) for future reference. If the patient is willing to participate, the RA will seek immediate written informed consent to participate in the trial (that is, trial consent) prior to their appointment with the GP. It is inherent in the nature of brief opportunistic advice that patients cannot be given warning of this or it ceases to be opportunistic.

We will ask to keep anthropometric and demographic data of patients who decline to participate in the trial. The researcher will record evidence of verbal consent for this on the Trial Screening Form.

#### Participant recruitment rate

We anticipate nine adults will consult per GP per session, of whom at least 25% will be obese and 75% of these will agree to participate in the study. In reality, the population of consulters is likely to be biased towards people with obesity because of its association with chronic disease, so the 25% is likely to be an underestimate. In trials of brief intervention for smoking cessation, recruitment rates (where they could be assessed) were high (100% [32], 93% [33], 88% [34], 89% [35], 87% [36], 96% [37], 92% [38], 90% [39]). As nearly all patients who are obese believe they have a weight problem and 84% would welcome GP support to manage this [25], we believe that 75% uptake is achievable. These figures would require us to attend 1,054 sessions and, with the staffing available, this could be achieved in 46 weeks to obtain the sample size of 1,824. Each GP will have intervened for about six sessions, which appears reasonable. The approach outlined here with the RA ‘in the waiting room’ was the most common approach used in other trials of brief interventions.

#### Randomization of participants

Participants will be randomized to a treatment arm at the baseline visit, which will be conducted at their own GP practice (see Measurement of outcomes [Visit 1, Baseline] for details). The RA will give eligible patients who are willing to participate in the trial an opaque A4 sealed envelope, which will contain a color-coded randomization card. The envelopes will be numbered in sequence. We will use block randomization of randomly sequenced blocks of 2 and 4 stratified by GPs, which will balance individual consulting styles. The trial statistician will be responsible for the production of randomization schedule, prior to recruitment.

The RA will ask patients to give the sealed envelope to their GP upon entering their consultation. The card inside the sealed envelope will reveal to the GP, but not the patient, which intervention to deliver. We will ask GPs not to open the randomization envelope until they are satisfied that it is suitable for the patient to be included in the trial (that is, after they have addressed the patient’s primary reason for visiting them and feel that it would be clinically appropriate to offer a brief intervention at this time to this patient - see Exclusion criteria above; once the randomization envelope is opened then the patient has been enrolled in the trial. It would undermine the ability of the study to detect differences between the arms if we enrolled participants who did not receive an intervention. Attached to the outside of each randomization card envelope will be a detachable ‘patient withdrawal card’. The RA will discretely write the patient’s weight and BMI on the back of the withdrawal card before handing the sealed envelope to them so that the GP will be aware of their up-to-date measurements, if required, during the consultation. Furthermore, there will be a tick box option on the card for GPs to select if they did not randomize the patient in to the trial, indicating that the person has not been randomized and therefore not enrolled. The tick box will indicate the reason for non-enrolment (that is, clinically inappropriate, not appropriate in the consultation or special reasons). If ‘special reasons’ is selected, the GPs will be asked to state why, if they can without breaking patient confidentiality, in the space provided. The sealed envelope and withdrawal card would be returned to the RA by the patient after their consultation (see Measurement of outcomes [Visit 1, Baseline]) and the envelope will then be replaced at the top of the randomization envelope pile for use by the next appropriate potential participant.

To examine the fidelity of the intervention, the brief-intervention component of a proportion of consultations by each GP will be audio-recorded (see Treatment providers below). If an audio recording is required, a card will be attached to the randomization card inside the randomization envelope reminding the GP to turn on the audio-recorder at the appropriate time during the consultation. If a patient has not consented to audio-recording of the brief intervention, the GP will be alerted to this by a sticker on the randomization envelope.

#### Unblinding

Due to the nature of this trial, it is necessary for the GPs and RAs to be aware of the patient’s treatment group allocation to ensure the correct intervention is provided. Thus, these personnel cannot be blinded to treatment. Patients and the members of the research team that will conduct follow-up assessments, however, will be blinded to treatment allocation.

As this is an open label trial the issue of unblinding the clinicians caring for the patient does not arise.

#### Withdrawal criteria

##### 

**Trial withdrawal** Participation in the trial is based upon patients consenting to take part. At the time of consent (see Recruitment of participants above) participants will be given a PIS to keep, which will provide details of what they should do if they no longer want to take part in the trial. The PIS will state that participants are free to withdraw from the trial at any point without it affecting their care and that they would not be contacted again by the research team. The PIS will include a telephone number for participants to contact the researchers for this or other reasons. Patients who have withdrawn will not be replaced but we will use their data up to the point that they withdrew unless they request that we do not do so. There are no withdrawal criteria other than patient or GPs request to withdraw.

##### 

**Treatment withdrawal** The main treatment is the offer of advice or support for weight loss from the GP. Participants cannot withdraw from receiving these interventions as they are a natural part of the consultation. Of course, it is possible that participants may choose not to return to the GP for further discussion of their weight. They may also choose not to attend a weight management intervention and, having initially attended, may choose not to complete a course of treatment. Such decisions will have no affect on the follow-up of participants in the trial.

### Definition of end of study

The end of study is the date of the last date of follow-up for the last patient. The appropriate standard operating procedure (SOP) (Primary Care Clinical Trials Unit [PC-CTU] SOP TM23), outlining the necessary procedures for trial closure, as written by the Clinical Trials & Research Governance Office, University of Oxford (CTRG), will be adhered to.

### Interventions

#### Intervention arms

After GPs have dealt with the patient’s original presenting complaint(s), they will briefly deliver one of two treatments, which the participant is randomly allocated to. The treatments include:

#### An ‘assistance-orientated’ treatment (intervention)

The GPs assistance-orientated treatment (that is, practical support) is aimed to last no more than a few seconds: “Did you know that the best way to lose weight is to go to Slimming World? I can refer you now for free on the NHS if you would like.” The choice of weight management service will mainly be determined by availability in the local area. In the Lighten Up trial, WW, RC, and SW each performed better than the control group [13].

If the person agrees to referral to the CWMS, the GP will ask the participant to make an appointment at reception to return in a month: “I know it can be difficult to lose weight, so I’d like you to return in a month to see how you’re getting on”. If the participant wants to try weight loss without assistance, the GP might say: “It’s fine for you to try to lose weight on your own but I know it’s hard. Would you make an appointment to return in a month to see how you are getting on?” This provides an opportunity to re-refer those who accepted referral but did not attend, refer those who tried to lose weight on their own but did not do well, prescribe orlistat to those who have followed the treatment program but not succeeded (in line with NICE guidance), and it lets the patient know their doctor is taking this seriously [30]. If the participant wants to discuss this with their GP, the GP will encourage the participant to make another appointment to do so (English GPs have 10 minutes per consultation).

Outside the context of the trial, we would envisage that the GP would make a referral to the weight management service in the same way s/he makes referrals to other services. We could envisage a system of direct booking, where the availability and days/times of local services are shown online on the GPs computer. However, because referral to weight management is uncommon, our GPs and patient representatives advised us it would be better to ask the research team to do this. We will aim for the RA in the practice to hold details of local groups that are available and make a booking immediately, to capitalize on the patient’s impulse to act. If this is not possible, we will arrange to telephone the participant at the earliest convenience with details of the options available.

The commercial weight management referral scheme typically consists of 12 vouchers enabling patients to attend classes for free (or use the online equivalent for anyone who does not want to or cannot attend groups). These services encourage weight loss via a healthy lifestyle such as increased physical activity, a reduced fat diet and offer group counseling for motivation and behavior change, with target setting for weight, activity, and energy intake. Intervention group participants will get 12 free sessions but are usually encouraged by the group leader to continue their involvement by paying, although in practice a minority do so. (We will obtain data on length of enrolment in the service from service providers and also from participants at follow-up.) Re-referral will be allowed within the rules operating in the PCT. Full details of the behavioral change techniques used by the three common services are available in the Lighten Up trial protocol [13].

#### An ‘advice-orientated’ treatment (control)

After considering the difficulties of a no-intervention control arm, we propose an advice-orientated treatment (that is, medical advice) such as: “It is important to lose weight because it would reduce your chances of getting heart problems, diabetes, and arthritis”*.* Other than no intervention, this appears to be the most common brief intervention offered currently and there is evidence that it is associated with attempts to lose weight. We believe it is polite and respectful and patients seem to find it helpful that their doctor passes on this kind of advice [25].

### Treatment providers

As noted above, the primary treatment (that is, delivery of either an assistance- or advice-oriented intervention) will be made by the participant’s GP. All GPs will receive trial specific training prior to commencing the study at their practice. We will train GPs using academic detailing methods, an established evidence-based technique for changing behavior [40]. The principal method of delivery will be via video-tutorials designed specifically for this trial, which will be available to GPs on a dedicated website before and during the trial. Each tutorial will be available to be viewed independently, rather than all having to be viewed at once, and also as downloadable audio files for alternative convenience. A similar approach has recently been established for smoking cessation (see http://www.NCSCT.co.uk/VBA); we intend to adopt a similar model.

The training video will consist of eight modules, lasting up to 2 hours in total: 1) the case for intervening in obesity; 2) the BWeL trial; 3) medical advice arm; 4) practical support arm part 1: the brief intervention; 5) practical support arm part 2: the review session at 1 month; 6) medical advice arm: the dedicated weight consultation; 7) practical support arm: the dedicated weight consultation; and 8) summary. The modules will be designed to address perceived barriers of the GPs to intervene in weight management. These barriers are that weight management is ineffective, which we will address by showing evidence that health risk is continuously associated with degree of overweight and that reduced weight reduces risk, even if the visible change is imperceptible. The barriers will also be addressed by evidence on the effectiveness of the commercial providers in the NHS referral schemes. We will also aim to address the lack of knowledge and hence confidence to intervene on weight management. This will be addressed firstly by reassuring GPs that we are not expecting them to undertake nutritional and physical activity assessments, and that the prime aim of the review session is reweighing, which is probably a key component of a weight loss intervention [30], and offer of referral/re-referral to the weight management services. Nevertheless, we recognize that patients might ask their GP for specific weight loss advice and our GP advisors were struggling to say more than “eat less, do more”*.* The modules will train GPs to deflect detailed questions about nutrition. We will provide GPs with a self-help guide published by the British Heart Foundation called ‘So You Want To Lose Weight…For Good’. We are not aiming to get GPs involved in behavioral change strategies or give particular or detailed advice, only allay concerns that might arise from offering patients a 10-minute review to discuss weight management. If there is any uncertainty about treatment delivery (for example, intervention content and methods of delivery) then GPs will be able to contact the research team for clarification.

To examine the fidelity of the interventions, we will seek the permission of both participants and GPs to audio-record the brief intervention component of a proportion of consultations (n = 5) by each GP. Most GPs will be familiar with recording their consultations as part of their training and most patients agree to this. To examine for evidence of consultation distortion, we will take the first 30 participants who have been recorded and compare the immediate post-consultation ratings of helpfulness and appropriateness to those not recorded. If evidence of distortion is found, we will not continue the recordings. Fidelity in the intervention group will be assessed by recording whether or not an offer of referral was made and whether or not there was supporting discussion (for example, encouragement to attend, advice on the superiority of the service over trying alone). Fidelity in the control group will be assessed on whether advice was given that linked weight loss to improved health and that no offer of referral to weight management was made.

Participants in the assistance-orientated intervention (that is, practical support) group will be offered referral to an evidence-based CWMS available in the NHS; for example, SW or RC. Immediately after the GP appointment, RAs at the GP practice will provide the intervention group participants with their weight management referral packs and assist booking the participant’s first appointment. Group leaders of these CWMS undertake rigorous weight management training through their organization and are expected to follow standard procedures.

### Safety

There is no reason to assume that this study will lead to an excess of adverse events. The treatment consists of advice from the GP and offer of referral to a weight management service, neither of which seem likely to create harm. We are aiming to recruit participants who are not specifically motivated to lose weight and therefore intend to keep participant burden to a minimum. Therefore, we will not monitor the occurrence of adverse events by trial arm.

We recognize that follow-up of participants requires us to behave sensitively and that, in a trial of this size, some participants will suffer serious health events or other misfortunes that will come to light in a follow-up telephone call. To ensure that future follow-up attempts are appropriate, we will log these events in the critical events log of the case report form (CRF).

### Statistics and analysis

#### Number of participants

The primary outcome will be weight change at 12 months. The following figures assume a 70% follow-up achieved in the Lighten Up trial and use the (conservative) ITT BOCF figures. We assume that 30% in the CWMS arm will take up referral and achieve the mean loss seen in our trial of the same intervention at 1 year (3.46 kg with BOCF for missing outcome data) and that 10% in the control arm will try to lose weight and achieve the mean loss seen in the Lighten Up trial (1.16 kg). The Prospective Studies Collaboration showed that obese people observed over a few years lose a little weight on average, at about 50 g/year [2]. These assumptions give mean weight loss of 1.07 kg versus 0.16 kg. The standard deviation for weight change was about 6.0 kg in both our Lighten Up and the Jebb trial [14,15]. On these assumptions, with 90% power and 5% type I error, we would need to randomize 912 people to each arm: 1,824 in total.

### Description of statistical methods

#### Primary outcome

The primary outcome of change in weight at 12 months will be analyzed by ITT BOCF for missing data. We will calculate mean and standard deviations of weight change at 12 months and compare the intervention arm with the control using analysis of covariance (ANCOVA) adjusting for baseline weight. This will be the primary analysis for the trial. A pre-specified sub-group analysis will examine the effect of gender, age (as a continuous variable), ethnic group, deprivation score derived from postcode and BMI. Residual plots will be used to examine model assumptions and where necessary transformations and/or bootstrapping will be used to address gross departure from normality. The possibility of GP effects will be explored via a random effects coefficient in the model.

#### Secondary outcomes

The difference in mean weight change at 3 months will be compared between the two treatment arms using ANCOVA adjusting for baseline weight with BOCF for missing data. Proportions of participants who achieve at least 5% or 10% weight loss at 12 months will be reported using descriptive statistics and the difference between two arms and 95% CI will be calculated. The costs of the two interventions will summarized as £ per kg and £ per kg/m^2^.

#### Non-efficacy outcomes

For participant’s reaction (assessed by two Likert scale questions) to the GP discussion of weight management when they visited for reasons other than their weight, total score will be calculated and compared between two arms using a t-test or a Mann–Whitney-U test if the data are too far from normality. The median for each question will be calculated and compared using a Mann–Whitney-U test.

Whether participants take any actions to manage their weight at 3 months and between 3 and 12 months following a brief opportunistic intervention reported by participants as a yes/no variable will be presented as frequencies and compared between the two arms using a chi-square test. What actions participants took will be coded as a three-category variable (that is, effective action, some action and no action) and compared for the difference between arms using proportional odds logistic regression.

Reactions of GPs to raising the issue of weight management opportunistically and giving the two types of brief intervention will be summarized by presenting the frequencies of each response to question 1, 2 and 7 of GP post-trial questionnaire part 2 in a bar chart. Total score will be calculated by summing questions 3 and 4 for the reaction of GPs to advise to lose weight and summing questions 5 and 6 for the reaction of GPs to offering support. These two total scores will be compared using a paired t-test.

Only summary statistics will be presented by intervention arms for the following non-efficacy outcomes:

• Change from pre-trial to post-trial in the frequencies of each response to the first 11 questions of GP questionnaire part 1 and the mean percentage for question 12 regarding GP attitudes to making opportunistic interventions and treating obese people.

• Frequency of each category of reasons for not trying to lose weight given by participants.

• Total number of CWMS sessions taken.

The qualitative data on responses to the intervention (as identified in Data handling and record keeping below) will be analyzed using framework analysis. This approach is a relatively quick method of qualitative enquiry that allows deductive exploration based on the aims and objectives of the interview. A thematic framework for analysis will be constructed prior to interview and unanticipated themes arising during interview will be added to the framework as appropriate. Data will be sorted and summarized under theme and subtheme headings from which the range and diversity of attitudes and experience will be summarized and explanatory linkages explored.

#### Other

Fidelity of the intervention will be assessed using a rating scale for key elements in the brief intervention types and summarized as a score (see Treatment providers section above for details of data). These are descriptive process data and will not be compared by arm.

A trial statistical analysis plan will be drawn up before the recruitment starts.

### Ethics

#### Participant confidentiality

The identification data of patients will be required for the registration process. The study coordination centre will preserve the confidentiality of all data obtained which are to be kept by the BWeL research team in compliance with the Data Protection Act (DPA) 1998 and PC-CTU SOP DM 01 04 “Data Management”, 23-03-2012; this includes data of trial participants. No personal identification data will be kept for patients who are eligible but refuse to participate; the research team will only keep their non-identifiable data to compare the characteristics of those who are eligible and do take part with those who are and do not. The trial management team will monitor confidentiality and consent will be sought from patients for the study sponsor or their delegates, members of the study team, regulatory authorities and the PCT to have direct access to patient medical records. All data obtained from patients who are not eligible to participate in the study will be provided to the GP practice only; the BWeL research team will not keep any of these data as they are not needed.

### Other ethical considerations

#### Ethics approval

The study co-ordination centre obtained approval from the National Research Ethics Service (Reference 13/SC/0028). The study will be conducted in accordance with the recommendations for physicians involved in research on human subjects adopted by the 18^th^ World Medical Assembly, Helsinki 1964 and later revisions.

#### Patient consent

See Recruitment of participants section above for details.

### Data handling and record keeping

#### Data handling, record keeping and retention

The trial is being run as part of the portfolio of trials in the PC-CTU. The data management will be run in accordance with the trials unit SOPs, which are fully compliant with the DPA and Good Clinical Practice (GCP). The source documents for the study will be the Trial Screening Form and Participant Contact Sheet (see Visit 1, above), CRFs and questionnaires, which will be securely held in locked, fire-protected storage facilities. These will be transferred from the site of the research visits to the university, and consent will explicitly be sought from participants to do so. The study database will be securely held and maintained by the PC-CTU. On completion of the trial and data cleaning, the study documentation, including patient identifiable information will be transferred to a secure, GCP compliant, external archiving facility, where they will be held either until the end of the trial and then destroyed or indefinitely after the end of the trial to allow participants to be recruited into further studies or additional follow-up, and patients will be asked to consent to either of these options. The database will be made anonymous and a secure compact disc containing the link between identification number and patient identifiable information will be stored in a secure archiving facility.

### Data access and quality assurance

Data will be kept in accordance with the DPA. The SOPs of the trials unit will be followed, which are designed to protect patient confidentiality. Patient identifiable information will be available to the person conducting follow-up as it is important that these data are known to them. Otherwise, confidentiality will be maintained and no-one outside the study team will have access to either the CRFs or the database.

### Case report forms

The secure online database will incorporate an online CRF; however, there will also be a paper copy of CRFs. In the context of the GP surgery it will not be possible to use an online CRF. In this case the paper CRF will be completed and data will be copied to the online version at a later date. As previously mentioned, paper CRFs will be securely held in locked, fire-protected storage facilities.

### Study management

The day-to-day management of the study will be coordinated by the University of Oxford research team; researchers will be provided with study specific training and be able to contact the research team for clarification.

### Study personnel

See Additional file 1 for study personnel details.

### Trial steering committee

A Trial Steering Committee (TSC) will be convened to provide overall supervision of the trial and ensure its conduct is in accordance with the principles of GCP and the relevant regulations. The TSC will agree the trial protocol and any protocol amendments; any amendments to the protocol will be made in accordance with the PC-CTU SOP TM SO_P9 “Protocol Amendments” 27-02-2012. Furthermore the TSC will provide advice to the investigators on all aspects of the trial. The TSC will be chaired by Professor Mike Lean, (Head of Department of Human Nutrition at the University of Glasgow). Rod Taylor (Associate Professor at Penninsula Medical School), Sarah Hardcastle (Senior Lecturer at the University of Brighton), Dr Isabelle Mantella (Clinical Lecturer at the University of Birmingham) and Mr and Mrs Fletcher (patient representatives who used the Lighten Up service) have agreed to be members. Dr Ghada Zoubiane (Programme Manager, Medical Research Council) has also agreed to join the committee.

### Data monitoring committee

We do not propose that a data monitoring and ethics committee would be useful, based on examples from http://www.ct-toolkit.ac.uk/ as this is an unblinded study with no substantial risk and no early termination rules. The final decision was made by the TSC, who agreed that it would not be useful for this trial and that they would adopt the role if required.

### Quality control and quality assurance procedures

The study will be conducted in accordance with the current approved protocol, ICH GCP, relevant regulations and PC-CTU SOPs. The PC-CTU has in place procedures for assessing risk management for trials which will outline the monitoring required. The monitoring will be carried out by the PC-CTU Quality Assurance Manager or equivalent. The investigators and all trial-related site staff will receive appropriate training in GCP and trial procedures.

Regular monitoring will be performed according to ICH GCP. Data will be evaluated for compliance with the protocol and accuracy in relation to source documents where possible. Following written SOPs, the monitors will verify that the clinical trial is conducted and data are generated, documented and reported in compliance with the protocol, GCP and the applicable regulatory requirements.

The PC-CTU Trial Management Committee (TMC) will be responsible for the monitoring of all aspects of the trial’s conduct and progress and will ensure that the protocol is adhered to and that appropriate action is taken to safeguard participants and the quality of the trial itself. The TMC will be comprised of individuals responsible for the trial’s day-to-day management (for example, the Chief Investigator, trial manager, statistician, data manager) and will meet regularly throughout the course of the trial.

### Financing and insurance

#### Funding

The Medical Research Council (National Prevention Research Initiative – Phase 4) is funding this study.

### Participant payments

We will compensate those participants who attend their 12 month follow-up appointment at their GP practice; a small honorarium fee (£10.00) to cover the cost and inconvenience of attending.

### GP payments

GPs will be paid, through NHS service support costs, for their time to deliver the intervention, which currently is not part of standard routine. These reimbursements are on a scale determined by the Primary Care Research Network.

### Negligent harm

The University has arrangements in place to provide for harm arising from participation in the study for which the University is the Research Sponsor.

### Non-negligent harm

The University has arrangements in place to provide for non-negligent harm arising from participation in the study for which the University is the Research Sponsor.

### Publication policy

We will present the results to the TSC prior to publication. The Investigators will be involved in reviewing drafts of the manuscripts, abstracts, press releases and any other publications arising from the study. Members of the TSC will be listed and their contribution acknowledged, as will the funding source (the Medical Research Council NPRI – Phase 4). Authorship will be determined in accordance with the ICMJE guidelines and other contributors will be acknowledged. The funders have no contribution to make on decisions on publication.

## Discussion

The aim of this trial is to test the effectiveness of a brief, opportunistic intervention for weight management in primary care; to our knowledge, no trials to date have investigated this issue. If successful, trial results could make the case for brief interventions for obese people consulting their GP and introduce widespread simple treatments akin to the NHS Stop Smoking Service. Likewise, the intervention could be introduced in the QOF and influence practice worldwide.

Throughout this protocol we identified some challenges that we envisaged we might encounter whilst undertaking the trial, and how we proposed dealing with them. Such challenges included: participant identification and recruitment; training GPs about the importance of weight management and how to intervene for the purposes of this trial; assessing the fidelity of the interventions; and ways to ensure high patient follow-up rates. Here we briefly discuss a few of the key challenges that the research team have encountered since the trial started; we believe this information will provide valuable insight for future trials of this nature, and especially about weight management in primary care.

### Timely completion of GP training

In a number of cases, GPs have failed to start and/or complete the online trial specific training prior to commencing the study at their practice. As a result, we have had to delay the start of the study in their practice and ensure that the training is fully completed by a rearranged date. As highlighted in the Treatment providers section above, GP training is delivered via a trial-specific online training video; the video consists of eight tutorials that are designed to address the perceived barriers of GPs to intervening in weight management. Each tutorial can be viewed independently, rather than all having to be viewed at once, and are also downloadable as an audio file for alternative convenience. The total length of the training video is up to 2 hours.

GPs felt that they did not have sufficient time to undertake the training, and advised that they had to address ‘more important’ issues within ‘spare’ practice time. Some GPs had also started but not completed the training; they apparently felt slightly overwhelmed by the detail and length of the first tutorial. As a result, the research team asked practice managers at the site initiations to warn GPs of this and to book a slot in their diary for them to complete the training. This solved the problem in new practices.

### Fidelity checks of intervention delivery

As highlighted in the Other ethical considerations section above, we examine the fidelity of a proportion of each GP session via audio-recordings to check that interventions are being delivered to protocol and in accord with that randomly allocated. The audio-recordings have been invaluable, far more than anticipated in fact, as we have identified fundamental errors by many GPs. For example, GPs forget key elements of the intervention, GPs offer additional interventions to participants in the control arm, and some GPs describe the research study (the content of the advice given in both arms of the trial) before delivering the intervention that the participant was randomly allocated to; this appears to occur more when GPs are new to research. The recordings have allowed us to pick up these problems and coach GPs to deliver the interventions to protocol early in their involvement in the trial and while the researcher and GP are in practice together. Subsequent recordings of the same GPs demonstrate that this feedback and coaching has been successful and rectified previous mistakes. Furthermore, prior to GPs commencing their initial BWeL sessions, the researchers now undertake a brief question and answer session with them to check their understanding of what they need to do, confirm their understanding of how the two interventions differ, check how they would respond to common scenarios and clarify any concerns they may have. Clarification and advice is given where needed.

### Practical issues for the recruiters in practice

As detailed in the Recruitment of participants section above, researchers conduct a screening session for patients visiting the participating GP while they are waiting for their appointment to measure body weight, height and percentage body fat. These measurements are essential to identify eligible patients for the trial. However, it soon became apparent that some mainly elderly patients had problems even undertaking the screening because their frailty and unsteadiness meant they could not be easily weighed or have their body fat assessed. In that context, it seemed probably inappropriate to offer weight loss advice anyway leading to exclusion on clinical grounds after seeing the GP. There was some discussion as to whether an age limit should be introduced to prevent situations of this kind. We decided originally against this and reaffirmed our decision not to use an upper age limit as it was frailty not age that was the issue. However, we initiated a ‘common sense’ rule to make life more comfortable for the researchers doing the screening “Could the patient be weighed accurately or not?” If a patient is unstable on the scales or has to be supported, then we cannot get an accurate measure and therefore we cannot establish whether they are eligible and screening need not be attempted or can be quickly aborted. These patients are not counted in numbers of people screened as they could not be measured accurately. Where researchers have concerns about a patient’s frailty the researcher now politely directs them to accept not being screened. Our observation is that many patients want to please, so if we make it clear that we are very happy not to weigh them they are happy with that.

Finally, regular monitoring of all operating procedures and discussions with the research team have ensured that if and when things do not happen as planned, then we can act upon them quickly by designing and implementing suitable changes and whilst it still can be corrected.

## Trial status

Participant recruitment began in June 2013.

## Abbreviations

ANCOVA: Analysis of covariance; BMI: Body mass index; BOCF: Baseline observation carried forward; BWeL: Brief intervention for weight loss; CRF: Case report form; CTRG: Clinical trials & research governance office, University of Oxford; CWMS: Commercial weight management services; DPA: Data protection act; GCP: Good clinical practice; GP: General practitioner; ITT: Intention-to-treat; MI: Motivational interviewing; NHS: National health service; OR: Odds ratio; PCT: Primary care trust; PIL: Participant information leaflet; PIS: Patient information sheet; QOF: Quality and outcomes framework; RA: Research assistant; RC: Rosemary Conley; PC-CTU: Primary care clinical trials unit; RCT: Randomized controlled trials; SOP: Standard operating procedure; SW: Slimming world; TMC: Trial management committee; TSC: Trial steering committee; WW: Weight watchers.

## Competing interests

PAv and AL have received hospitality from Weight Watchers on two occasions. Slimming World and Rosemary Conley have agreed to fund free treatment courses for NHS patients participating in the trial. SJ has benefited from research grants to her institution from Weight Watchers. In a personal capacity she writes a nutrition column for the Rosemary Conley Diet and Fitness magazine and is a member of the Tanita Medical Advisory Board. She has received hospitality from Weight Watchers on two occasions. DL is running a randomized controlled trial in which the intervention is provided free of charge in the form of Slimming World membership vouchers. The other authors declare that they have no competing interests.

## Authors’ contributions

AL and PAv designed the study and drafted the manuscript. All authors (AL, KJ, PAd, AD, AF, SJ, DL, SC, AC, JJ, BT and PAv) contributed to study design, manuscript revisions and read and approved the final manuscript.

## Supplementary Material

Additional file 1 Study personnel sheet. Click here for file
