# Supplementary material for: A brief intervention for weight management in primary care: study protocol for a randomized controlled trial
Source: Trials. 2013 Nov 19;14:393. doi: 10.1186/1745-6215-14-393 (PMC3842709; doi:10.1186/1745-6215-14-393)
Supplement: Additional file 1 — Study personnel sheet. [file 1745-6215-14-393-S1.docx]

**Appendix A. Study personnel sheet**

**Brief Study Title:** Brief interventions for Weight Loss (BWeL)

**Study Start Date:** 1^st^ September 2012

| **Personnel** | **Name** | **Address** | **Telephone** | **E-mail** |
| --- | --- | --- | --- | --- |
| **Sponsor** | Ms. Heather House | The University of Oxford, Joint Research Office, Block 60, Churchill Hospital, Old Road, Headington, OX3 7LE | 01865 572224 | ctrg@admin.ox.ac |
| **Chief Investigator** | Prof. Paul Aveyard | Primary Care Health Sciences, The University of Oxford, Radcliffe Observatory Quarter,  Woodstock Road, Oxford, OX2 6GG | 01865 617860 | paul.aveyard@phc.ox.ac.uk |
| **Principal Investigator (project)** | Dr. Amanda Lewis | Primary Care Health Sciences, The University of Oxford, Radcliffe Observatory Quarter,  Woodstock Road, Oxford, OX2 6GG | 01865 617871 | amanda.lewis@phc.ox.ac.uk |
| **Trial Co-Ordinator** | Miss Sarah Clarke | Primary Care Health Sciences, The University of Oxford, 2^nd^ Floor 23-38 Hythe Bridge Street, Oxford, OX1 2ET | 01865 617958 | Sarah.clarke@phc.ox.ac.uk |
| **Researcher Assistant** | Miss Anna Christian | Primary Care Health Sciences, The University of Oxford, 2^nd^ Floor 23-38 Hythe Bridge Street, Oxford, OX1 2ET | 01865 617963 | Anna.christian@phc.ox.ac.uk |
| **Recruitment Assistant** | Mrs Janet Robertson | Primary Care Health Sciences, The University of Oxford, 2^nd^ Floor 23-38 Hythe Bridge Street, Oxford, OX1 2ET | 01865 617845 | janet.robertson@phc.ox.ac.uk |
| **Recruitment Assistant** | Miss Parvati Herman-Howe | Primary Care Health Sciences, The University of Oxford, 2^nd^ Floor 23-38 Hythe Bridge Street, Oxford, OX1 2ET | 01865 617194 | Parvarti.herman-howe@phc.ox.ac.uk |
| **Administrator** | Miss Kathryn Hood | Primary Care Health Sciences, The University of Oxford, 2^nd^ Floor 23-38 Hythe Bridge Street, Oxford, OX1 2ET | 01865 617963 | Kathryn.hood@phc.ox.ac.uk |
